# Estimating employment from energy-efficiency investments

**DOI:** 10.1016/j.mex.2020.100955

**Published:** 2020-06-08

**Authors:** Marilyn A. Brown, Anmol Soni, Yufei Li

**Affiliations:** School of Public Policy, Georgia Institute of Technology, 365 Cherry Street, Atlanta, GA 30332, USA

**Keywords:** Clean energy jobs, Employment impacts, Green jobs, Input-output analysis

## Abstract

We develop a methodology for estimating the number and types of jobs that would result from investments in energy efficiency in homes, businesses, and industry. The methodology involves the development of input-output (I-O) bills of goods that characterize how energy-efficiency funds would be spent across sectors of the economy. The methodology builds on and adds greater articulation to the research conducted in prior studies of U.S. energy-efficiency policies.

• The first two steps involve estimating the magnitude of investments in energy-efficient technologies and systems required to produce a unit of energy consumption reduction, and then identifying how these investments are expensed across the broad investment categories, which creates the preliminary “bills of goods” for investments in energy efficiency in homes, businesses, and industry.
• The third step involves soliciting feedback on the preliminary bills of goods from experts in delivering and evaluating energy-efficiency programs, and then making necessary modifications.
• In the final step we apply the input-output coefficients representing the bills of goods to estimate the direct, indirect and induced employment per million dollars of investment in energy efficiency.

## Highlights

• Uses input-output approach to estimate clean energy jobs from investments.
• Method for estimating the number and types of jobs from energy-efficiency investments.
• Estimates jobs from efficiency investments in homes, businesses, and industry.
• Creates “bills of goods” to examine the job impacts of investments in energy efficiency.

## Graphical abstract

Comparison of Employment Multipliers Across Energy Sectors (FTE/$million investment)(Note: Total values are correct; the sums of components as shown may not add to the totals, due to rounding.)

**Figure:**
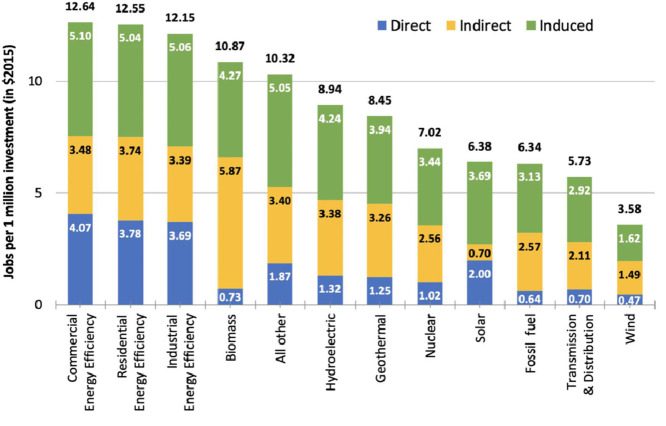

Specifications Table

**Table:** 

Subject Area:	Energy
More specific subject area:	*Energy efficiency*
Method name:	*Input-Output Estimates of Clean Energy Jobs*
Name and reference of original method:	*Clean energy “bills of goods”*
Resource availability:	*Climate and Energy Policy Lab, Georgia Institute of Technology*

## Method details

*[Methodological protocols should be in sufficient detail to be replicated. There is no word limit! You can include figures, tables, videos – anything that you feel will help others to reproduce the method. The main focus of the paper should be on the technical steps required for this method, more than results; where appropriate, guide the reader through the procedure and provide all extra observations or ”tricks” alongside the protocol. Results and Discussion are not sections included in the MethodsX format. However, providing data that validate the method is valuable and required. This section could become a “method validation” paragraph within the Method Details section.]*

Prior work estimating employment from energy-efficiency investments comprises a wide range of studies, across different types of technologies, sectors of the economy, and scales. Typically, these studies rely on an input-output modeling approach to estimate the macroeconomic impacts, including employment generation. The employment impacts can be categorized into direct, indirect and induced. The direct effects relate to sectors that get affected by direct economic activity due to higher investment through various programs. Indirect effects primarily include the materials and industry demand as a second order effect. Finally, induced effects reflect the increased spending on consumer goods and services by those earning higher incomes due to the direct and indirect effects across the economy.

As the world grapples with a pandemic with devasting effects on health systems, consumer spending, and the entire U.S. economy, with widespread shutdowns causing significant economic retrenchment, there are major consequences in store for our energy systems. These may challenge the ability of energy economy models of historic U.S. conditions as used in this study to provide robust forecasts of employment from energy efficiency investments in the future.

In the short-term, the reduction in industrial activity and closure of non-essential industries is likely to reduce investments in energy and related upgrades. One exception could be the expanded use of residential energy as the result of stay-at-home orders. “EIA assumes, in particular, that household usage of electronic equipment such as computers and televisions will increase. Other uses of electricity, such as for cooking and for heating water, may also rise. Household use of air conditioning during the summer months is also likely to be higher than normal as more people stay home during the daytime.”^1^

In the medium-term, as federal stimulus investments revive the global economy and household spending, the clean energy sector may gain some additional spending as witnessed in the 2008–09 recession. Legislators and stakeholders are already seeking funding for clean energy technologies in Coronavirus stimulus packages.^2^ The International Energy Agency and many other cleantech advocates recommend that clean energy be put at the heart of stimulus plans to counter Covid-19, which could cause a resurgence in energy-efficiency investments, perhaps with altered patterns of investment that are yet to be determined.^3^

## Literature

Bell et al. [3] provide an evaluation of different methodologies used to measure job creation in energy efficiency improvements. The authors summarize the prevailing studies as following one of two approaches – bottom-up where surveys and interviews are used to generate the number of jobs in the sector, and top-down approaches where economic modeling (such as computable general equilibrium, input-output, econometric models) is used to estimate the macroeconomic effect of investments in clean energy. The authors also describe a combination of the two major types, i.e. hybrid approaches where top-down and bottom-up analyses are combined.

The US Energy and Employment Report [13] estimated that in 2016 there were nearly 2.2 million jobs in the energy efficiency sector. By 2018, energy efficiency jobs had grown to 2.35 million jobs [11]. More than half (1.3 million) of these employees work in the construction industry; others work in the design and manufacturing of products, and the delivery of professional services. In the report on Energy Efficiency Jobs in America, Environmental Entrepreneurs and E4TheFuture [7] find that jobs in the energy efficiency industry tend to be concentrated in smaller business, with 25 or fewer employees. Further, these companies focus primarily on installation, trade and distribution related aspects of the industry.

The latest World Employment and Social Outlook by the International Labor Organization deploys an I-O modeling approach to estimate the employment impacts of sustainability [10]. Similarly, the report by Pollin et al. [12] examines the net implications of expanded investments in clean energy and energy efficiency by using the IMPLAN I-O model [9]. The authors find the net effects of rising clean energy investments and falling share of fossil fuels lead to an increase in the total jobs generated over two decades. The job growth is primarily because clean energy investments are more “labor intensive” and require a larger share of “domestic content”. Garett-Peltier [8] takes a similar approach, examining the net employment effects of redirecting fossil fuel investments towards clean energy. Incorporating a similar input-output analysis into the results or a computable general equilibrium analysis of carbon taxes, Brown et al. [4] estimate significant employment growth from energy-efficiency investments. The novel application of input-output analysis to energy efficiency, in combination with general equilibrium modeling, is the subject of this Methods-X paper.

Looking across metropolitan areas, Yi [14] uses an econometric approach to examine the employment effects of clean energy policies. He finds that overall, each clean policy adopted for the sector leads to a 1% increase in the number of green jobs. At a local scale, DeShazo et al. [6] also examine the wide range of clean energy programs in the Los Angeles County and use an I-O model to assess the actual expected job impacts. The authors find that more than 16 job-years could be created for every million dollars invested in these programs operated by the Los Angeles Department of Water and Power. And finally, in the evaluation of Maryland's EMPower energy efficiency program, Baatz and Barrett [1] estimate a total of 2000 jobs were generated in 2011.

## Methodology and approach

Several models are available to analyze employment impacts, including ACEEE's DEEPER model and NREL's JEDI model. However, for this study we first deployed Georgia Tech's version of the National Energy Modeling System (NEMS), the premier and arguably most influential U.S. energy modeling tool. NEMS data is more up-to-date than the data in DEEPER, and it better represents energy-efficiency investments compared with JEDI. The model uses an I-O approach to calculate employment across 49 sectors of industry and services. The Macroeconomic Activity Module Documentation provides detailed information on industrial classification and employment calculations.^4^

Preliminary analysis identified a gap in the NEMS employment calculations. It was detected empirically when the estimates showed a small net loss of U.S. jobs through 2030, despite large-scale investments in energy efficiency, which is a labor-intensive activity. Examination of the NEMS architecture, and subsequent discussions with EIA NEMS modelers revealed that the investments in energy efficiency are not recycled back to the IHS Global Insights macroeconomic model that estimates GDP and employment. As a result, NEMS underestimates the employment and GDP impacts of scenarios that model energy-efficiency incentives. It does not include the direct, indirect, and induced jobs generated by the incremental expenditures on high-efficiency equipment and materials. These overlooked jobs were then estimated through independent input-output (I-O) modeling, and the results added to the NEMS employment estimates to provide a more complete assessment.

**Fig. 1:**
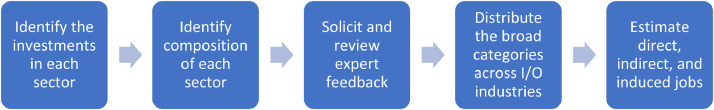
Approach to calculating additional jobs from I/O data.

To estimate the employment impact of investments in energy efficiency, we develop an approach that applies employment coefficients from the IMPLAN input-output model to results from the National Energy Modeling System (NEMS) (Fig. 1).

### Step 1: identify the investments in each sector

**Fig. 2:**
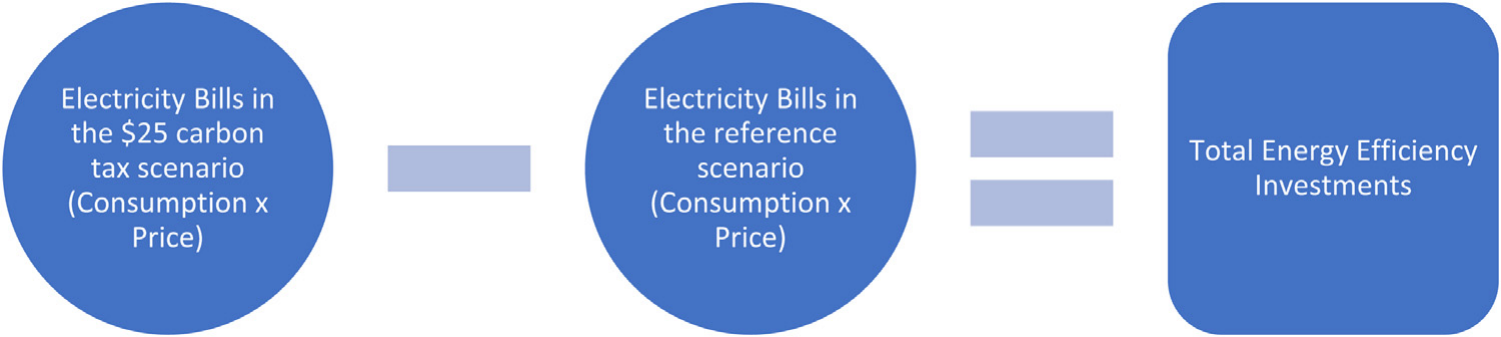
Approach to calculating step 1: energy efficiency investments.

The first step estimates investments in energy efficiency technologies and systems required to produce the energy consumption reductions. As noted earlier, the NEMS model predicts large scale improvements in energy efficiency as a result of a $25/ton carbon tax. However, in order to calculate the monetary value of the investments, we calculated the difference in electricity bills in the two cases. Since the energy consumption in all three sectors (residential, commercial and industry) is expected to go down but the prices are expected to increases, utility revenues will likely increase, thus spurring additional investments in energy efficiency. The energy efficiency investments are assumed to be equivalent to the value of the energy saved in each (as would occur in an on-bill financing program). This involved estimating the energy consumption, prices and bills in the $25 carbon tax scenario modeled in NEMS, by year and census region. For this, we first calculate the change in electricity consumption and prices. We multiply these to arrive at the bills for households and the resulting change in energy-efficiency jobs (Fig. 2). The results are presented in Table 1.

**Table 1:** Derivation of energy-efficiency job estimates of a $25 carbon tax.

		Reference Case			$25 Carbon Tax			$25 Carbon Tax – Reference Case			
		Electricity Consumption (a)(TWh)	Prices (b) (cents/kWh)	Electricity Bills *c*=axb(Billion 2017$)	Electricity Consumption (d)(TWh)	Prices (e)(cents/kWh)	Electricity Bills *f*=dxe(Billion 2017$)	Change in Electricity Consumption (d-a) (TWh)	Change in Prices (e-b) (cents/kWh)	Change in Bills (f-c) (Billion 2017$)	Change in Energy-Efficiency Jobs(Thousand)
Residential	2020	1408.9	13.4	188.3	1395.9	14.5	202.0	−12.9	1.1	13.7	**172.3**
	2025	1389.1	13.8	192.1	1346.7	15.5	209.3	−42.4	1.7	17.2	**216.2**
	2030	1409.4	14.0	197.9	1362.3	15.8	215.3	−47.0	1.8	17.4	**218.4**
	2035	1434.7	14.1	201.8	1375.3	16.2	222.2	−59.3	2.1	20.4	**256.7**
	2040	1471.0	14.1	207.1	1400.1	16.4	230.0	−71.0	2.4	22.9	**288**
	2045	1506.5	14.0	210.9	1425.1	16.7	237.4	−81.3	2.7	26.5	**332.5**
	2050	1545.6	13.9	215.4	1450.7	16.8	243.9	−94.9	2.9	28.5	**357.8**
Commercial	2020	1374.0	11.1	151.9	1365.1	12.1	165.2	−8.9	1.0	13.3	**166.8**
	2025	1397.3	11.2	156.9	1363.4	12.8	175.0	−33.9	1.6	18.1	**227.4**
	2030	1417.5	11.3	160.6	1377.8	12.9	178.4	−39.7	1.6	17.8	**223.7**
	2035	1443.4	11.2	162.1	1390.1	13.1	182.3	−53.3	1.9	20.3	**254.3**
	2040	1481.4	11.2	165.6	1412.2	13.3	187.3	−69.2	2.1	21.8	**273**
	2045	1533.4	11.0	168.9	1446.4	13.3	192.9	−87.0	2.3	24.0	**301**
	2050	1614.5	10.9	175.7	1506.6	13.3	200.2	−107.9	2.4	24.5	**307.1**
Industrial	2020	1023.7	7.3	75.1	995.5	8.8	87.8	−28.2	1.5	12.7	**108.7**
	2025	1099.0	7.4	81.5	1053.9	8.9	93.7	−45.1	1.5	12.2	**152.8**
	2030	1139.9	7.5	85.0	1077.4	9.0	97.4	−62.5	1.6	12.4	**149.8**
	2035	1165.6	7.4	86.3	1081.7	9.2	99.6	−83.9	1.8	13.3	**164.8**
	2040	1200.7	7.4	88.7	1097.2	9.3	102.3	−103.6	1.9	13.6	**170.8**
	2045	1226.7	7.3	89.8	1099.8	9.5	104.9	−126.9	2.2	15.1	**187.7**
	2050	1246.2	7.3	91.0	1097.2	9.6	105.6	−149.0	2.3	14.5	**183.6**

Note: Changes in prices are correct; they may differ from “e-b”, as shown, due to rounding.

### Step 2: identify composition of each sector

The second step involves distributing these investments across the broad investment categories. This step creates the “bills of goods” that characterize how energy efficiency expenditures are spent. This follows an approach similar to that of Baer et al. [2] and Garrett-Peltier [8], which focus on industry spending patterns. We build on the prior works as we combine the results of the NEMS (general equilibrium approach) and somewhat incomplete recycling of revenues in the NEMS I-O model by superimposing the energy-efficiency job gains from an external I-O model in IMPLAN. Bills of goods are available for many green energy systems such as solar and wind, but they are not well defined for energy efficiency. To estimate the effects of energy-efficiency investments in each sector, we created separate bills of goods for each of the three major end-use sectors: residential, commercial, and industrial. For simplicity, we assume that energy-efficiency expenditures are spent similarly across the census regions.

### Step 3: solicit and review expert feedback

The third step is to seek expert opinion on these estimates and validating the bill of goods created in Step 3. Draft bills of goods were developed by the authors and reviewed by a group of energy-efficiency experts in the Southface Energy Institute, ACEEE, the Greenlink Group, Georgia Public Service Commission, University of Massachusetts, Sterling Energy and Independent Consultants. As a result of the experts’ feedback, we incorporated the following changes:

○ Increased the allocation to construction in residential and commercial sectors – The reviewers’ feedback converged in that the new investments in energy efficiency will require additional construction spending. Responding to this, we increased the share of energy efficiency investment allocated to the sector.
○ Reduced the level of investment in Program Administration – In our initial distribution of investments, we had assumed that a significant share would be allocated towards the administrative expenses of government-led energy-efficiency programs. However, the experts suggested that the shares would be lower than our estimates and accordingly, we reduced the share of related categories in the final distribution.
○ Added a sector called Architecture and Engineering Services – Finally, one of the reviewers suggested including a new category given that some energy investments would also require changes to the current architectural and engineering approaches in building construction and design. Accounting for this suggestion, we added this category in our distribution. This also allowed us to redistribute some of the investments from the reduced share of “Program Administration”.

We implemented a two-step approach, following up with the experts once the first round of changes had been incorporated. No additional changes were suggested in the second round. The final distribution of the Bills of Goods is shown in Table 2.

**Table 2:** Summary of bills of goods for three energy-efficiency sectors.

	Residential	Commercial	Industry
Construction	20%	20%	9%
HVAC&R	20%	20%	7%
Water heating	6%	5%	3%
Lighting	10%	10%	5%
Material for envelope	12%	7%	6%
Motors, drives and back-up generators	0%	3%	10%
Other electrical equipment	5%	5%	12%
Industrial machinery manufacturing	0%	0%	16%
Energy and environmental management and smart controls	15%	18%	20%
Insurance and finance	2%	2%	2%
Program administration	5%	5%	5%
Architecture and engineering services	5%	5%	5%
Total	100%	100%	100%

### Step 4: distribute the broad categories across IMPLAN sectors

The fourth step uses the IMPLAN I-O coefficients to estimate the direct, indirect and induced employment per $1 million of investment. The shares from Step 3 were further decomposed to reflect the specific industries covered in the IMPLAN software. The software lists 536 industries reflecting the first 3-digits from the North American Industry Classification System (NAICs). This detailed breakdown is presented in Table 3, Table 4, Table 5. The tables summarize the bills of goods for all three energy-efficiency sectors. These tables have four columns – the first represents the broad spending category as noted in Table 2. The next column reflects the IMPLAN industry description/name as provided within the software. The next two columns are the shares we attribute to the sub-sector and the aggregate across the broad sector identified in the first column.

**Table 3:** Bills of goods for residential energy efficiency.

Sector	Description	Sub-sector shares	Sectoral shares
Construction	Construction of new single-family residential structures	5.00%	20%
	Construction of new multifamily residential structures	5.00%	
	Construction of other new residential structures	4.00%	
	Maintenance and repair construction of residential structures	2.50%	
	Manufactured home (mobile home) manufacturing	1.50%	
	Brick, tile, and other structural clay product manufacturing	2.00%	
HVAC&R	Air purification and ventilation equipment manufacturing	6.00%	20%
	Heating equipment (except warm air furnaces) manufacturing	5.00%	
	Air conditioning, refrigeration, and warm air heating equipment manufacturing	5.00%	
	Household cooking appliance manufacturing	1.00%	
	Household refrigerator and home freezer manufacturing	1.00%	
	Household laundry equipment manufacturing	1.00%	
	Other major household appliance manufacturing	1.00%	
Water heating	Plastics pipe and pipe fitting manufacturing	1.00%	6%
	Pottery, ceramics, and plumbing fixture manufacturing	1.00%	
	Iron, steel pipe and tube manufacturing from purchased steel	1.00%	
	Power boiler and heat exchanger manufacturing	1.00%	
	Plumbing fixture fitting and trim manufacturing	1.00%	
	Fabricated pipe and pipe fitting manufacturing	1.00%	
Lighting	Electric lamp bulb and part manufacturing	5.00%	10%
	Lighting fixture manufacturing	5.00%	
Material for envelope	Wood windows and door manufacturing	2.00%	12%
	Paint and coating manufacturing	2.00%	
	Polystyrene foam product manufacturing	2.00%	
	Urethane and other foam product (except polystyrene) manufacturing	2.00%	
	Mineral wool manufacturing	2.00%	
	Flat glass manufacturing	2.00%	
Other electrical equipment	Small electrical appliance manufacturing	1.00%	5%
	Power, distribution, and specialty transformer manufacturing	1.00%	
	Storage battery manufacturing	1.00%	
	Wiring device manufacturing	1.00%	
	All other miscellaneous electrical equipment and component manufacturing	1.00%	
Energy and environmental management and smart controls	Optical instrument and lens manufacturing	1.00%	15%
	Electronic computer manufacturing	1.00%	
	Computer storage device manufacturing	1.00%	
	Computer terminals and other computer peripheral equipment manufacturing	1.00%	
	Broadcast and wireless communications equipment manufacturing	1.00%	
	Other communications equipment manufacturing	1.00%	
	Bare printed circuit board manufacturing	1.00%	
	Semiconductor and related device manufacturing	1.00%	
	Printed circuit assembly (electronic assembly) manufacturing	1.00%	
	Other electronic component manufacturing	1.00%	
	Industrial process variable instruments manufacturing	1.00%	
	Analytical laboratory instrument manufacturing	1.00%	
	Data processing, hosting, and related services	1.00%	
	Computer systems design services	1.00%	
	Other computer related services, including facilities management	1.00%	
Insurance and finance	Insurance carriers	1.00%	2%
	Insurance agencies, brokerages, and related activities	1.00%	
Program administration	Federal electric utilities	1.25%	5%
	State government electric utilities	1.00%	
	Local government electric utilities	1.00%	
	* Employment and payroll of state govt, non-education	0.50%	
	* Employment and payroll of local govt, non-education	0.50%	
	* Employment and payroll of federal govt, non-military	0.75%	
Architecture and engineering services	Architectural, engineering, and related services	1.50%	5%
	Specialized design services	1.50%	
	Environmental and other technical consulting services	2%

**Table 4:** Bills of goods for commercial energy efficiency.

Sector	Description	Sub-sector share	Sectoral Shares
Construction	Construction of new health care structures	5.00%	20%
	Construction of new educational and vocational structures	5.00%	
	Construction of new commercial structures, including farm structures	10.00%	
HVAC&R	Air purification and ventilation equipment manufacturing	5.00%	20%
	Heating equipment (except warm air furnaces) manufacturing	5.00%	
	Air conditioning, refrigeration, and warm air heating equipment manufacturing	10.00%	
Water heating	Plastics pipe and pipe fitting manufacturing	0.50%	5%
	Fabricated pipe and pipe fitting manufacturing	0.50%	
	Iron, steel pipe and tube manufacturing from purchased steel	0.50%	
	Plumbing fixture fitting and trim manufacturing	1.50%	
	Power boiler and heat exchanger manufacturing	2.00%	
Lighting	Electric lamp bulb and part manufacturing	5.00%	10%
	Lighting fixture manufacturing	5.00%	
Material for envelope	Flat glass manufacturing	1.00%	7%
	Metal window and door manufacturing	0.25%	
	Plastics material and resin manufacturing	0.25%	
	Adhesive manufacturing	0.50%	
	Fabricated structural metal manufacturing	0.25%	
	Mineral wool manufacturing	1.25%	
	Polystyrene foam product manufacturing	1.00%	
	Urethane and other foam product (except polystyrene) manufacturing	1.25%	
	Spring and wire product manufacturing	0.50%	
	Blind and shade manufacturing	0.25%	
	Valve and fittings, other than plumbing, manufacturing	0.25%	
	Sheet metal work manufacturing	0.25%	
Energy and environmental management and smart controls	Optical instrument and lens manufacturing	1.00%	18%
	Electronic computer manufacturing	1.00%	
	Computer storage device manufacturing	1.00%	
	Computer terminals and other computer peripheral equipment manufacturing	1.00%	
	Broadcast and wireless communications equipment manufacturing	1.00%	
	Other communications equipment manufacturing	1.00%	
	Bare printed circuit board manufacturing	1.00%	
	Semiconductor and related device manufacturing	1.00%	
	Printed circuit assembly (electronic assembly) manufacturing	1.00%	
	Other electronic component manufacturing	1.00%	
	Industrial process variable instruments manufacturing	1.00%	
	Analytical laboratory instrument manufacturing	0.50%	
	Data processing, hosting, and related services	0.50%	
	Computer systems design services	0.50%	
	Automatic environmental control manufacturing	4.50%	
	Other computer related services, including facilities management	1.00%	
Motors, drives and back-up generators	Speed changer, industrial high-speed drive, and gear manufacturing	1.00%	3%
	Fluid power pump and motor manufacturing	1.00%	
	Motor and generator manufacturing	1.00%	
Other electrical equipment	Power, distribution, and specialty transformer manufacturing	1.00%	5%
	Storage battery manufacturing	1.00%	
	Wiring device manufacturing	1.00%	
	Small electrical appliance manufacturing	1.00%	
	All other miscellaneous electrical equipment and component manufacturing	1.00%	
Insurance and finance	Insurance carriers	1.0%	2%
	Insurance agencies, brokerages, and related activities	1.0%	
Program administration	Federal electric utilities	1.25%	5%
	State government electric utilities	1.00%	
	Local government electric utilities	1.00%	
	* Employment and payroll of state govt, non-education	0.50%	
	* Employment and payroll of local govt, non-education	0.50%	
	* Employment and payroll of federal govt, non-military	0.75%	
Architecture and engineering services	Architectural, engineering, and related services	1.50%	5%
	Specialized design services	1.50%	
	Environmental and other technical consulting services	2%

**Table 5:** Bills of goods for industrial energy efficiency.

Sector	Description	Sub-sector share	Sectoral Shares
Construction	Construction of new manufacturing structures	5.0%	9%
	Construction of new power and communication structures	4.0%	
HVAC&R	Air purification and ventilation equipment manufacturing	2.0%	7%
	Industrial process furnace and oven manufacturing	1.5%	
	Heating equipment (except warm air furnaces) manufacturing	1.5%	
	Air conditioning, refrigeration, and warm air heating equipment manufacturing	2.0%	
Lighting	Electric lamp bulb and part manufacturing	2.5%	5%
	Lighting fixture manufacturing	2.5%	
Material for envelope	Wood windows and door manufacturing	1.5%	6%
	Paint and coating manufacturing	0.8%	
	Polystyrene foam product manufacturing	0.8%	
	Urethane and other foam product (except polystyrene) manufacturing	1.0%	
	Mineral wool manufacturing	1.0%	
	Sheet metal work manufacturing	1.0%	
Water heating	Plastics pipe and pipe fitting manufacturing	0.5%	3%
	Concrete pipe manufacturing	0.5%	
	Iron, steel pipe and tube manufacturing from purchased steel	0.5%	
	Power boiler and heat exchanger manufacturing	1.0%	
	Fabricated pipe and pipe fitting manufacturing	0.5%	
Motors, drives and back-up generators	Turbine and turbine generator set units manufacturing	3.5%	10%
	Mechanical power transmission equipment manufacturing	3.5%	
	Motor and generator manufacturing	3.0%	
Other electrical equipment	Small electrical appliance manufacturing	3.0%	12%
	Power, distribution, and specialty transformer manufacturing	3.5%	
	Storage battery manufacturing	1.5%	
	Wiring device manufacturing	1.5%	
	All other miscellaneous electrical equipment and component manufacturing	2.5%	
Industrial machinery manufacturing	Speed changer, industrial high-speed drive, and gear manufacturing	3.0%	16%
	Pump and pumping equipment manufacturing	3.0%	
	Air and gas compressor manufacturing	2.5%	
	Conveyor and conveying equipment manufacturing	2.5%	
	Welding and soldering equipment manufacturing	2.0%	
	Fluid power cylinder and actuator manufacturing	2.0%	
	Fluid power pump and motor manufacturing	1.0%	
Energy and environmental management and smart controls	Optical instrument and lens manufacturing	1.0%	20%
	Electronic computer manufacturing	2.0%	
	Computer storage device manufacturing	2.0%	
	Computer terminals and other computer peripheral equipment manufacturing	2.0%	
	Broadcast and wireless communications equipment manufacturing	1.0%	
	Other communications equipment manufacturing	1.0%	
	Bare printed circuit board manufacturing	1.0%	
	Semiconductor and related device manufacturing	2.0%	
	Printed circuit assembly (electronic assembly) manufacturing	1.0%	
	Other electronic component manufacturing	0.5%	
	Automatic environmental control manufacturing	1.0%	
	Industrial process variable instruments manufacturing	0.5%	
	Analytical laboratory instrument manufacturing	1.0%	
	Data processing, hosting, and related services	1.0%	
	Computer systems design services	1.5%	
	Other computer related services, including facilities management	1.5%	
Insurance and finance	Insurance carriers	1.0%	2%
	Insurance agencies, brokerages, and related activities	1.0%	
Program administration	Federal electric utilities	1.25%	5%
	State government electric utilities	1.00%	
	Local government electric utilities	1.00%	
	* Employment and payroll of state govt, non-education	0.50%	
	* Employment and payroll of local govt, non-education	0.50%	
	* Employment and payroll of federal govt, non-military	0.75%	
Architecture and engineering services	Architectural, engineering, and related services	1.50%	5%
	Specialized design services	1.50%	
	Environmental and other technical consulting services	2%	

Note: sectoral shares are correct; they may differ from the sum of the sub-sector shares as shown, due to rounding.

It is interesting to note here that the indirect and induced effects surpass the direct effects in all cases. Across the three energy-efficiency sectors, the jobs multipliers are highest in the commercial sectors, which is a function of the way spending is distributed across different sub-sectors (Table 2). The spending in smart management and controls is higher in the commercial and industrial Sectors than in the residential. Further, spending on materials is a smaller share of the total investments in the sector.

This distribution was then used to calculate the spending in each industry and the consequent implications on total additional jobs generated.

**Fig. 3:**
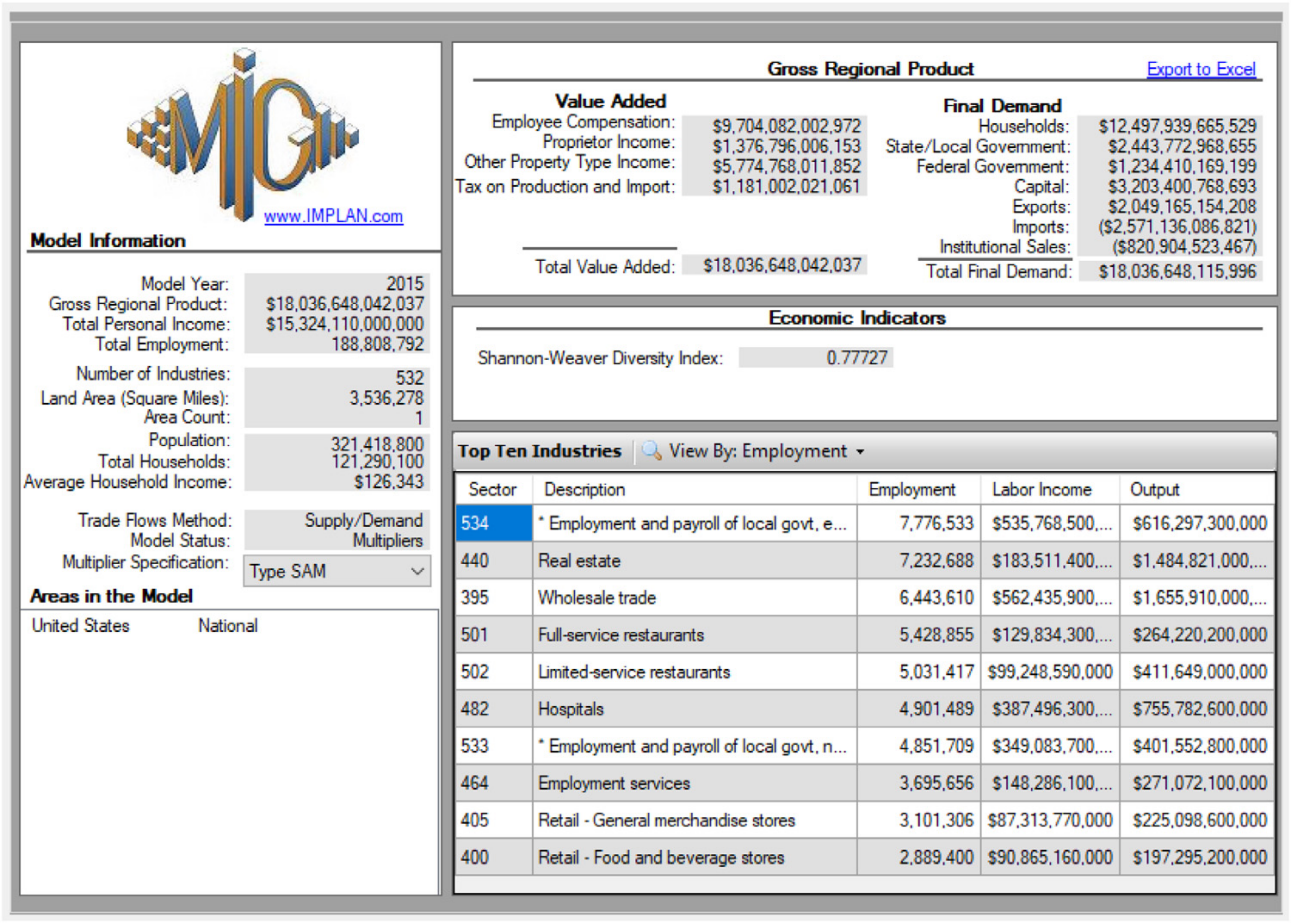
IMPLAN 3.0 interface.

We used IMPLAN version 3.0 for generating the multipliers (Fig. 3).

## Employment estimates

The employment multipliers for investments in energy supply (based on IMPLAN-defined sectors) are lower than for investments in energy efficiency (Table 6). As noted earlier, using the I-O model allows us to disaggregate the total employment effects into Direct, Indirect and Induced effects. Taking the example of investments in industrial energy efficiency here, as per Table 6, an additional spending of $1 million increases the total jobs in sectors affected by higher investments in Table 5 creates 3.69 additional FTE jobs. Additionally, each sector that benefits directly also generates second-order effects in sectors that provide raw materials and support series to the sector. For example, as a result of spending in the construction of new structures (Row 1 in Table 5), jobs will be generated in sectors that produce construction materials. Such second order effects constitute the indirect effects. In our example of industrial energy efficiency, the indirect jobs account for 3.39 additional FTE jobs per million dollars of investment. Finally, the increased employment and resultant wages in the hands of direct and indirect beneficiaries will lead to an increase in consumption spending in sectors such as restaurants, hotels etc. leading to 5.06 additional induced jobs as a result of industrial energy efficiency spending.

**Table 6:** Comparison of employment multipliers across energy sectors (FTE/$million investment in $2015).

	Direct	Indirect	Induced	Total
Electric power generation*				
Wind	0.47	1.49	1.62	3.58
Transmission & distribution	0.70	2.11	2.92	5.73
Fossil fuel	0.64	2.57	3.13	6.34
Solar	2.00	0.70	3.69	6.38
Nuclear	1.02	2.56	3.44	7.02
Geothermal	1.25	3.26	3.94	8.45
Hydroelectric	1.32	3.38	4.24	8.94
All other	1.87	3.40	5.05	10.32
Biomass	0.73	5.87	4.27	10.87
Energy efficiency				
Industrial	3.69	3.39	5.06	12.15
Residential	3.78	3.74	5.04	12.55
Commercial	4.07	3.48	5.10	12.64

Note: total values are correct; the sums of components as shown may not add to the totals, due to rounding.

⁎ Source: IMPLAN Group [9].

To further illustrate the use of this methodology, we apply the multipliers to an analysis of the employment impact of implementing a $25 carbon tax on the U.S. economy starting in 2020 and escalating 5% each year. We use GT-NEMS to analyze the employment impacts on the energy supply-side economic activities. We then estimate the additional employment that would occur as the result of energy-efficiency jobs based on the bills of goods shown in Table 2 through Table 5.

**Fig. 4:**
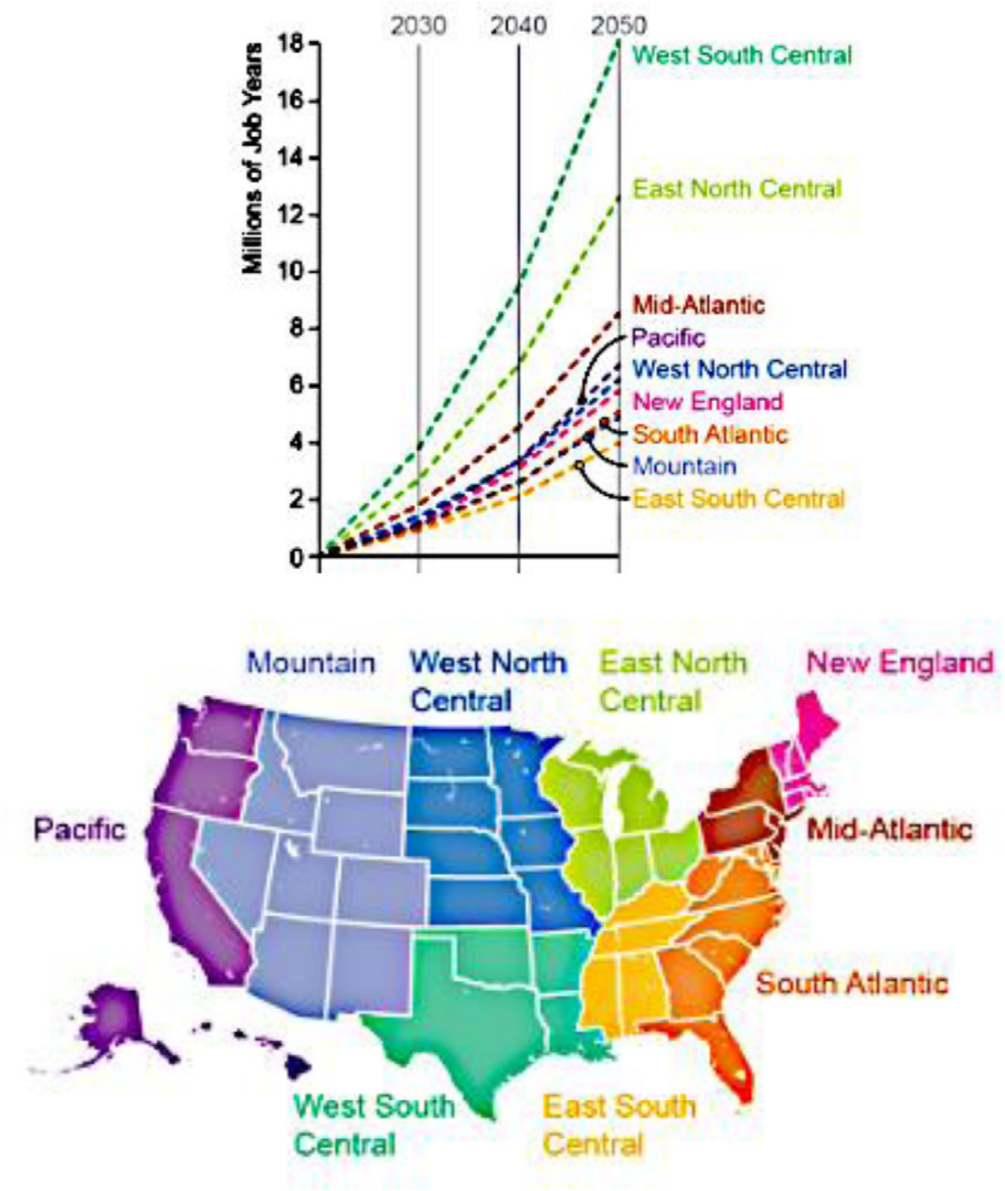
The cumulative difference in employment, by region, with a $25 carbon tax (figure created by authors using data published in [5]).

This methodology was used by Brown and Ahmadi [5] in their analysis of a $25 carbon tax, showing that a tax could boost U.S. employment significantly. If implemented in 2020, a $25 carbon tax could expand U.S. employment by 1.4 million jobs each year between 2020 and 2030, which is nearly a 1 percent increase above the baseline forecast of 160 million jobs in 2030. Altogether, an estimated 72 million “job years” would be created over the three decades with a $25 carbon tax. (Note that if one job continues after one year for another 12 months, it represents two job years.) Fig. 4 summarizes these results.

## Summary

**Fig. 5:**
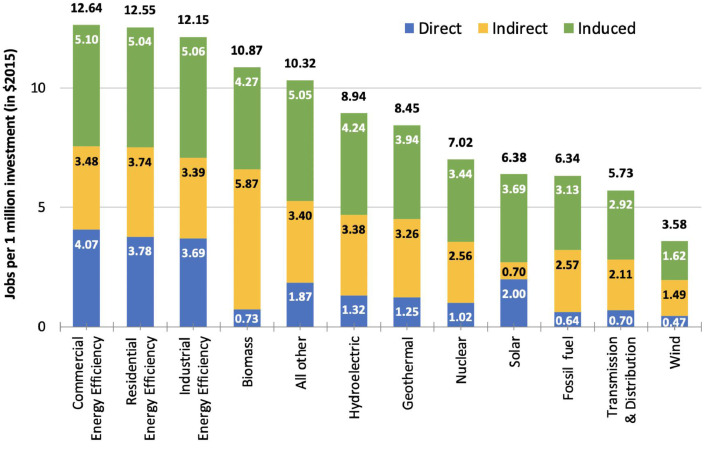
Comparison of employment multipliers across energy sectors (FTE/$million investment in $2015). (Note: total values are correct; the sums of components as shown may not add to the totals, due to rounding.).

Illustrations of how this methodology can be deployed are available in several recent publications [4,5,2]. Fig. 5 portrays our estimates of jobs per million dollars of investment (in $2015) across energy sectors of the U.S. economy, including three energy-efficiency domains.

## CRediT authorship contribution statement

**Marilyn A. Brown:** Conceptualization, Methodology, Investigation, Writing - original draft, Writing - review & editing, Visualization. **Anmol Soni:** Methodology, Investigation, Writing - original draft, Writing - review & editing, Visualization. **Yufei Li:** Data curation, Methodology, Investigation, Visualization.

## Declaration of Competing Interest

The authors declare that they have no known competing financial interests or personal relationships that could have appeared to influence the work reported in this paper.

## Footnotes

1 https://www.eia.gov/outlooks/steo/report/electricity.php.

2 https://www.greentechmedia.com /articles/read/clean-energy-groups-seek-tax-credit-extensions-direct-pay-provisions-in-coronavirus-stimulus-package.

3 https://www.iea.org/commentaries/put-clean-energy-at-the-heart-of-stimulus-plans-to-counter-the-coronavirus-crisis

4 https://www.eia.gov/outlooks/aeo/nems/documentation/macroeconomic/pdf/m065(2018).pdf

## References

[1] Baatz B., Barrett J. (2017). Maryland Benefits: Examining the Results of EmPOWER Maryland Through 2015.

[2] Baer P., Brown M.A., Kim G. (2015). The job generation impacts of expanding industrial cogeneration. Ecol. Econ..

[3] Bell C.J., Barrett J., McNerney M. (2015). Verifying Energy Efficiency Job Creation: Current Practices and Recommendations.

[4] Brown M.A., Li Yufei, Soni Anmol (2020). Are all jobs created equal? Regional employment impacts of a U.S. carbon tax. Appl. Energy.

[5] Brown M.A., Ahmadi Majid (2019). Would a green new deal add or kill jobs?. Sci. Am..

[6] DeShazo J.R., Turek A., Samulon M. (2014). Efficiently Energizing Job Creation in Los Angeles.

[7] Environmental Entrepreneurs (E2) & E4TheFuture, 2016. Energy Efficiency Jobs in America. https://www.e2.org/wp-content/uploads/2016/12/EnergyEfficiencyJobsInAmerica_FINAL.pdf

[8] Garrett-Peltier H. (2017). Green versus brown: comparing the employment impacts of energy efficiency, renewable energy, and fossil fuels using an input-output model. Econ. Model.

[9] IMPLAN Group, LLC (2017). IMPLAN 2017.

[10] International Labour Organization (ILO), 2018. World Employment and Social Outlook 2018: Greening With Jobs.

[11] National Association of State Energy Officials (NASEO) and Energy Futures Initiative (EFI), 2019. US Energy and Employment Report.

[12] Pollin R., Garrett-Peltier H., Heintz J., Hendricks B. (2014). Green Growth a US Program for Controlling Climate Change and Expanding Job Opportunities.

[13] U.S. EIA (2017). US Energy and Employment Report.

[14] Yi H. (2013). Clean energy policies and green jobs: an evaluation of green jobs in US metropolitan areas. Energy Policy.

